# Efficient modal analysis of plasmonic nanoparticles: from retardation to nonclassical regimes

**DOI:** 10.1515/nanoph-2021-0668

**Published:** 2022-02-02

**Authors:** Wei Yan, Min Qiu

**Affiliations:** Key Laboratory of 3D Micro/Nano Fabrication and Characterization of Zhejiang Province, School of Engineering, Westlake University, 18 Shilongshan Road, Hangzhou 310024, Zhejiang Province, China; Institute of Advanced Technology, Westlake Institute for Advanced Study, 18 Shilongshan Road, Hangzhou 310024, Zhejiang Province, China

**Keywords:** modal method, plasmonic nanoparticles, quantum effects of electrons

## Abstract

With recent developments in nanotechnologies, metal nanoparticles permeate a wide range of dimension scales, from light wavelength-scale domains down to a few nanometers approaching electronic scales. The electrodynamics at metal surfaces hosts a rich interplay between plasmon oscillations, retardation effects of light, and nonclassical (quantum) effects of electrons. Incorporating all these effects and modeling optical responses of nanoparticles generally rely on pure numerical methods, which are, however, disadvantageous in physical interpretations and computational speed. Herein, we establish a modal method that accurately predicts plasmon responses of metal nanoparticles, including both retardation and nonclassical corrections on an equal footing. The proposed method, based on electrostatic plasmon modes, is parameterized by a set of geometrically dependent factors, which, once computed, can be repeatedly used for same-shaped nanoparticles independent of size and material composition. The predictive accuracy of the method is examined for single nanoparticles, multi-scale plasmonic architectures—such as dimer structures with deep-nanometer gap—and geometrically deformed structures, with feature dimensions ranging from a few nanometers to hundreds of nanometers.

## 1 Introduction

Metal nanoparticles support surface plasmon resonances (SPRs), collective oscillations of free electrons at metal surfaces restored by induced electric fields [1]. In the electrostatic limit, SPRs are simply formulated as eigensolutions of Poisson’s equation in terms of electric potential (*ϕ*_*n*_, *n* = 1, 2, 3, …) [2–4]:

(1) $$\nabla \cdot \left[\Lambda_{n}+f\left(r\right)\right]\nabla \phi_{n}\left(r\right)=0.$$

Here *f*(**r**) is a filling function with a value of 1 inside the nanoparticle and 0 otherwise. The geometrical eigenvalue Λ_*n*_ determines the resonance frequency through its definition Λ_*n*_ ≡ *ɛ*_bg_/Δ*ɛ*(*ω*), where Δ*ɛ*(*ω*) = *ɛ*_np_(*ω*) − *ɛ*_bg_, i.e., the difference between the permittivities of the nanoparticle (np) and the background (bg).

The scale invariance of Poisson’s equation render SPRs a unique property: they localize electric fields free of scale constraints, e.g., light wavelength [1, 5]. This property does not exist in conventional phonotic resonances based on dielectric materials, which are limited by the diffraction limitation of light. Hence, metal nanoparticles are widely exploited in various technological/scientific scenarios that demand localized electric fields to enhance light-matter interactions, e.g., in surface-enhanced Raman sensing [6, 7], single photon emitters [8, 9] and nanomedicine [10]. As a result, SPRs form one of central concepts in nanophotonics.

**Figure 1:**
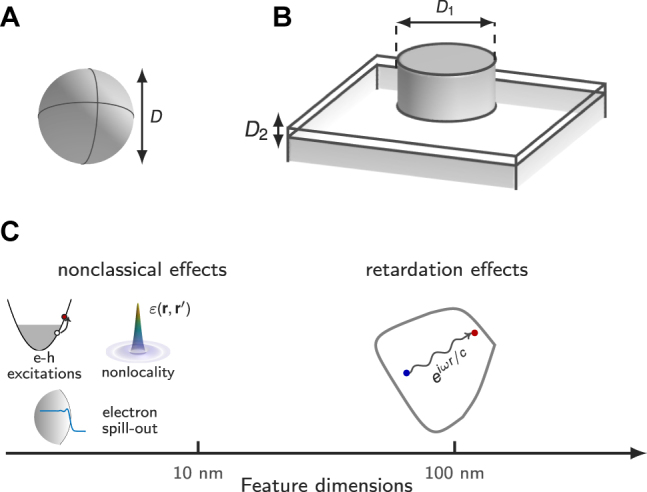
Nanoplasmonic particles, and their associated retardation and nonclassical effects. (A) Spherical particles of diameter *D* exhibit resonant redshift and line broadening as the size increases above tens of nanometers due to retardation effects of light. On the other hand, as the particle size decreases below 10 nm, nonclassical effects of electrons kick in and also lead to resonant shift and size-dependent damping. (B) Multi-scale architectures, such as film-coupled plasmonic particles, with particle size *D*_1_ comparable to light wavelength and gap size *D*_2_ close to electronic length scales, host noticeable retardation and nonclassical effects simultaneously. (C) Sketch of size-dependent retardation and nonclassical effects. Specifically, the nonclassical effects include three major contributions from (i) surface-enhanced e–h excitations, i.e., Landau damping; (ii) nonlocality of permittivity response functions; (iii) spill-out of conduction electrons outside the metal boundary.

The electrostatic SPRs constitute a natural basis for computing optical responses of plasmonic nanostructures [2, 11, 12]. Their unique advantage lies in their merely geometrical dependence, meaning that, the SPRs, belonging to a specific particle shape, can be repeatedly used independent of particle size and material composition. This property is absent in other modal basis, such as quasi-normal modes that are eigensolutions of Maxwell’s wave equation constrained by outgoing-wave conditions [13–15]. As a result, the modal method based on the static SPRs is advantageous in computational efficiency, besides of providing physical intuitions. However, its practical applicability is limited due the neglect of (i) the retardation effects of light and (ii) the nonclassical (quantum) effects of electrons (Figure 1) [16–18]. First, in major plasmonic-based applications, the SPRs are typically excited by far-filed illuminations to exploit enhanced light scattering. Therein, the efficient excitations of the SPRs require that particle size cannot be too small compared to light wavelength, which thus results in the noticeable retardation effects that are, however, absent in Eq. (1). Second, on the opposite end, the researchers nowadays are pursuing extreme light-matter interactions by reducing structure dimension to deep nanoscales (below 10–20 nm) [19–22]. The extremely localized SPRs can be excited by near-filed sources, such as electron beams and quantum emitters. In this regime, the intrinsic quantum-wave (nonclassical) nature of electrons onsets, whose comprehensive investigations bring the emerging field of quantum plasmonics (see Figure 1(C) for a classification of various nonclassical effects). Even more, exploring both advantages of efficient light coupling/scattering and extreme light confinement in the same platforms, multi-scale plasmonic architectures (Figure 1(B))—such as particle dimers and film-coupled nanoparticles, with feature dimensions covering both light and electron length scales—are intensively studied [23–25], highlighting rich interplay between plasmon oscillations, and the retardation/nonclassical effects.

Incorporating the retardation and nonclassical effects in plasmonic modeling is at the heart of theoretical explorations in nanoplasmonics. The successful schemes largely rely on coupling full Maxwell’s wave equation with semiclassical/phenomological models for electrons, such as the hydrodynamic Drude model [26–29] and the Feibelman *d*-parameter method [16, 21, 25, 30], [31], [32]. The simulations generally employ fully numerical approaches, e.g., with finite element method and boundary element method. However, they are computational expensive and cannot provide straightforward physical intuitions. On the other hand, there are a few papers attempting to integrate the retardation and nonclassical corrections into the modal framework of the electrostatic SPRs. Notably, in Reference [33], the authors started with the volume Green’s integral equation and established an analytical method that includes the retardation effects systematically. Complementarily, in Reference [32], the nonclassical effects are analytically treated in the electrostatic limit. However, a modal framework based on the electrostatic SPRs, incorporating both the retardation and nonclassical effects, is still absent and remains to be established and examined.

In this article, grounded on the electrostatic SPRs, we establish a modal method for simulating plasmonic nanoparticles. In the proposed method, the retardation effects of light and the nonclassical effects of electrons are treated on an equal footing by combining the previous insights from References [32, 33]. The method describes optical responses using a set of geometrical factors, which, once computed, can be repeatedly used for same-shaped nanoparticles independent of size and material composition. We validate the method for versatile plasmonic structures commonly accessed to the experiments, including single nanoparticles and multi-scale plasmonic architectures.

## 2 Theory formalism

### 2.1 Incorporation of retardation and nonclassical effects

Consider a plasmonic system (Figure 1(A) and (B)), which is driven by an incident electric field **E**_in_. We incorporate the nonclassical effects of electrons by introducing a surface polarization **P** ^*Q*^ source on the background side of the particle boundary ∂Ω [16, 25, 31]:

(2) $$P^{Q}\left(\omega ;r\right)\equiv \underset{\delta \to 0^{+}}{\lim}\;\Delta \varepsilon \left(\omega \right)\bar{\bar{d}}\left(\omega \right)\cdot E_{\text{tot}}\left(r-\delta \hat{n}\right).$$

Here, $\bar{\bar{d}}\equiv d_{⊥}\hat{n}\hat{n}+d_{∥}\left(\bar{\bar{I}}-\hat{n}\hat{n}\right)$ . *d*_⊥,∥_ are the so-called Feibelman *d*-parameters, which are the centroids of the induced charge and the normal derivative of the tangential polarization current, respectively. The use of the Feibelman *d*-parameters allows us to elegantly include three major nonclassical effects, that is, surface-enabled intraband electron–hole excitations through $Im\left(\bar{\bar{d}}\right)$ , nonlocality of permittivity and electron spill-out through $Re\left(\bar{\bar{d}}\right)$ . On the other hand, quantum tunneling [24] and size quantization [34] effects are outside the applicability of the *d*-parameters, which, nevertheless, only become important when feature sizes are below 1 nm.

The optical response of the system is represented by expanding the total electric field **E**_tot_ with the modal fields of the electrostatic SPRs [**E**_*n*_ ≡ −***∇*** *ϕ*_*n*_, see Eq. (1)]:

(3) $$E_{\text{tot}}=\underset{n}{\sum }a_{n}\left(\omega \right)E_{n}.$$

We emphasize that this expansion is approximate and only includes the curl-free component of **E**_tot_, i.e., assuming that **∇** × **E**_tot_ = 0. Nevertheless, as shall be validated below and also has been concluded in Reference [33], this approximation gives rather accurate results for particle size up to hundreds of nanometers as long as the retardation corrections are properly added.

We derive the modal coefficients |*a*⟩, $|a〉\equiv \left[a_{1};a_{2};\ldots \right]$ , by plugging the modal expansion Eq. (3) into the retarded Maxwell’s wave equation that includes the nonclassical surface polarization **P** ^*Q*^. This treatment automatically includes both the retardation and nonclassical effects. Next, we express the electric field **E**_tot_ with the Green’s function surface integral formalism, concentrating on the fields on the particle boundary ∂Ω (see Supplementary Section S1). Finally, the equation that determines |*a*⟩ is derived as (see Supplementary Section S2):

(4) $$\Lambda \left(\omega \right)|a〉=\left[H^{0}+H^{R}+H^{Q}+H^{R-Q}\right]|a〉+|S〉.$$

Here, |*S*⟩ $\left(|S〉\equiv \left[S_{1};S_{2};\ldots \right]\right)$ is the vector of the incident electric field projected into the SPR basis, with $S_{n}{=\oint }_{\partial \Omega }\phi_{n}\left(r\right)E_{\text{in}}\left(r\right)\cdot \hat{n}{\text{d}}^{2}r$ . $H^{0}=\Lambda_{n}\delta_{nm}$ with SPR eigenvalue Λ_*n*_ defined in Eq. (1). $H^{R}$ , $H^{Q}$ and $H^{R-Q}$ account for the corrections from retardation (*R*), quantum (*Q*), and coupled retardation-quantum (*R*–*Q*) effects, respectively. To reveal the geometrical dependencies of $H^{R,Q,R-Q}$ , we introduce the following dimensionless parameters [12]

(5) $$s^{R}\equiv k_{\text{bg}}L,\;{\bar{\bar{s}}}^{Q}\equiv \bar{\bar{d}}/L,$$

where *L* is a characteristic length of the particle and $k_{\text{bg}}\equiv \omega \sqrt{\varepsilon_{\text{bg}}}/c$ is the wavenumber of light in the background medium. The normalized modal fields relate to *L* according to

$$\underset{\partial \Omega }{\oint }\sigma_{n}\left(r\right)\phi_{m}\left(r\right)=\delta_{nm}L^{3},$$

where $\sigma_{n}\equiv \lim_{\delta \to 0^{+}}\frac{E_{n}\left(r-\delta \hat{n}\right)\cdot \hat{n}}{\Lambda_{n}}$ represents the surface charge of the *n*th mode, and *δ*_*nm*_ = 1 for *n* = *m* and otherwise 0. The matrix components of $H^{R,Q,R-Q}$ are then given by

(6a) $$H_{nm}^{R}=f_{nm;2}^{R}{\left(s^{R}\right)}^{2}+f_{nm;3}^{R}{\left(s^{R}\right)}^{3}+\cdots \;,$$

(6b) $$H_{nm}^{Q}={\bar{\bar{f}}}_{nm}^{Q}:{\bar{\bar{s}}}^{Q},$$

(6c) $$H_{nm}^{R-Q}=\left[{\bar{\bar{f}}}_{1}^{R-Q}s^{R}+{\bar{\bar{f}}}_{2}^{R-Q}{\left(s^{R}\right)}^{2}+\cdots \;\right]:{\bar{\bar{s}}}^{Q}.$$

Here, $f_{nm}^{R,Q,R-Q}$ are dimensionless geometrical factors, expressed by

$$\begin{array}{ll} f_{nm;k}^{R} & =-\frac{i^{k}}{4\pi \Lambda_{m}L^{k+3}}\underset{\partial \Omega }{\oint }\underset{\partial \Omega }{\oint }|r-r^{\prime }{|}^{k-1}\left[\hat{n}\left(r\right)\times E_{m}\left(r\right)\right]\\ & \;\;\;\;\;\;\;\;\;\cdot \left[\hat{n}\left(r^{\prime }\right)\times E_{n}\left(r^{\prime }\right)\right]{\text{d}}^{2}r^{\prime }{\text{d}}^{2}r,\\ {\bar{\bar{f}}}_{nm}^{Q} & =-\frac{\Lambda_{n}}{\Lambda_{m}L^{2}}\underset{\delta \to 0^{+}}{\lim}\underset{\partial \Omega }{\oint }E_{n}\left(r+\delta \hat{n}\right)⊗E_{m}\left(r-\delta \hat{n}\right){\text{d}}^{2}r, \end{array}$$

and ${\bar{\bar{f}}}_{nm;k}^{R-Q}$ is given in Eq. (S.2.9) in the SI.

Equations (4)–(6) determine the modal excitation coefficients, with which the optical responses of the system can then be reconstructed. They are derived by solving the Green’s function surface integral formalism [see Eq. (S.1.15) in the Supplementary Material], which characterizes the optical response of the system, with the SPR basis. The derivations are given in Supplementary Section S2. Specifically, Eq. (6a) gives the retardation corrections. The associated terms, $f_{nm;k}^{R}$ (*k* = 2, 3, …), are expressed with double surface integrals. Numerically, we find that the retardation corrections can be accurately computed by truncating the series to *k* = 5. Note that, in Reference [33], the retardation corrections are alternatively given by double volume integrals. In this regard, our surface-integral formulations are more computationally efficient. Moreover, the expression of $f_{nm;k}^{R}$ only involves electric fields on the particle boundary, thus being compatible with most existing SPR solvers that are implemented based on the surface-integral/boundary-element methods and solve boundary fields.

Equation (6b) gives the nonclassical correction in the absence of the retardation effects. Its diagonal terms involving ${\bar{\bar{f}}}_{nn}^{Q}$ describes the leading-order nonclassical corrections, and has been comprehensively studied in Reference [32]. Interestingly, as will be discussed later, by recognizing the geometrical meaning of the Feibelman *d*-parameters, this nonclassical correction can be straightforwardly generalized to account for geometrical deformations (perturbations) of the particle, thereby rendering the present formalism an even broader applicability. Equation (6c) characterizes the coupling between the retardation and nonclassical effects. Despite its complicated expression, this term is numerically found to be tiny and is omitted through our numerical examples.

### 2.2 Independent-mode approximation

By retaining a few dominantly excited modes in Eq. (4), the excited modal coefficients can be computed by solving a small matrix problem. Nevertheless, a further simplification can be made by discarding modal couplings enabled by the retardation and nonclassical effects. More precisely, we neglect the off-diagonal terms in $H^{R,Q,R-Q}$ , i.e., assuming that the independence of different electrostatic SPRs in the presence of the retardation and nonclassical effects. The approximation is accordingly termed as independent-mode approximation. Under this approximation, the excitation coefficients are given by

(7a) $$a_{n}≃\frac{S_{n}}{\Lambda \left(\omega \right)-\Lambda_{n}^{\prime }}.$$

with perturbed eigenvalue $\Lambda_{n}^{\prime }$ expressed as

(7b) $$\Lambda_{n}^{\prime }≃\Lambda_{n}+\underset{k=2,3,\ldots }{\sum }f_{nn;k}^{R}{\left(s^{R}\right)}^{k}+{\bar{\bar{f}}}_{nm}^{Q}:{\bar{\bar{s}}}^{Q}.$$

**Figure 2:**
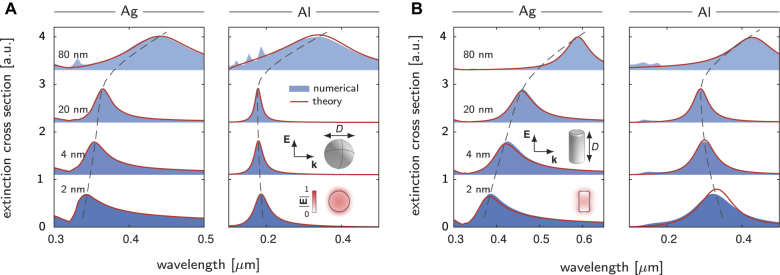
Modal analysis of extinction cross-section spectra for metal spheres (A) and rods (B), incorporating both retardation and nonclassical effects. The nanoparticles, made of Ag and Al and embedded in a dielectric medium with a refractive index of 1.33, are considered. The aspect ratio of the rods (diameter dived by height) in **B** is fixed to be 0.5. The results predicted by the modal method show quantitative agreements with the numerical solutions obtained with COMSOL Multiphysics. In the modal method, a single dipolar mode is retained with its excitation coefficient computed with Eq. (7a). The retardation series in Eq. (6a) is truncated to the power *k* = 5. The resonances show rich size-dependent shifts (dashed lines) and broadening due to the retardation and nonclassical effects. The insets sketch scattering of the nanoparticles by plane waves and profiles of dipolar modes.

**Figure 3:**
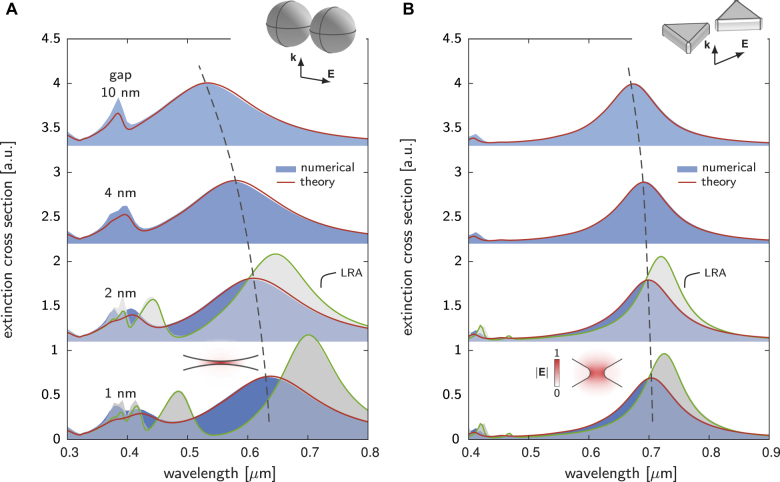
Modal analysis of extinction cross-section spectra for spherical dimers (A) and bow-tie structures (B), incorporating both retardation and nonclassical effects. The nanoparticles, made of Ag, are embedded in a dielectric medium with refractive index of 1.33. The gap varies from 10 nm (top) to 1 nm (bottom). The spheres in (A) have a diameter of 80 nm, while the triangles in (B) have a side length of 100 nm and a height of 25 nm. The results predicted by the modal method agree well with the numerical solutions obtained with COMSOL Multiphysics. In the modal method, four modes in both (A) and (B) are used, and the modal coefficients are obtained by solving Eq. (4). The retardation series in Eq. (6a) is truncated to the power *k* = 5. As the gap narrows progressively, plasmon hybridizations become stronger, which renders the resonances a redshift (visualized by dashed lines). To illustrate the effects of the nonclassical effects at small gap sizes, we include the extinction cross-section spectra under the local-response approximation (LRA) by setting *d*_⊥_ = 0. The insets sketch scattering of the dimer structures by plane waves and profiles of the lowest-order bounding dipolar resonances.

As shown in the results below, the independent-mode approximation gives rather accurate results for single nanoparticles (Figure 2). In relation with two closely related publications, References [32, 33], Eq. (7b) represents a straightforward generalization that its pole gives complex resonance frequencies including the leading-order corrections due to the retardation and nonclassical effects. For complicated plasmonic structures, such as dimer structures, this approximation loses its predictive accuracy, and the inclusion of the modal couplings due to the retardation and nonclassical effects is recommended (Figure 3).

### 2.3 Nonclassical effects and geometrical deformation

**Figure 4:**
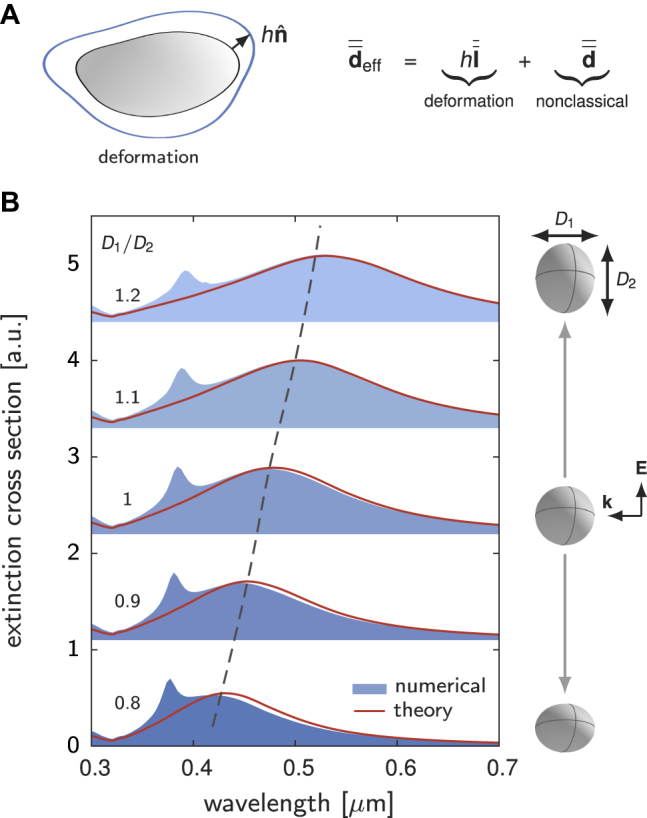
Modal analysis of geometrically deformed nanoparticles. (A) Optical response of a deformed nanoparticle can be effectively represented by introducing a generalized *d*-parameter, ${\bar{\bar{d}}}_{\text{eff}}$ . (B) Extinction cross-section spectra of Ag spheroids embedded in a dielectric medium with a refractive index of 1.33. In the modal method, a single dipolar mode (polarized in the direction of the incident electric field) of the sphere is used to predict the extinction spectra of spheroids with *D*_1_ = 100 nm and *D*_2_/*D*_1_ varied from 0.8 to 1.2. The retardation series in Eq. (6a) is truncated to the power *k* = 5.

We observe that the nonclassical surface polarization, Eq. (4), is formally equivalent to the effective surface polarization used to represent geometrical deformations (perturbations) of a nanoparticle. More specifically, consider that a nanoparticle is geometrically deformed with its boundary shifted by a vector $h\left(r\right)\hat{n}$ (see Figure 4(A)). To the leading order in the deformation, the optical responses of the deformed particle can be represented by placing a surface polarization $P^{D}\left(\omega ;r\right)=\lim_{\delta \to 0^{+}}\Delta \varepsilon \left(\omega \right)h\left(r\right)E_{\text{tot}}\left(r-\delta \hat{n}\right)$ on the particle boundary of the original particle [35]. Comparing **P** ^*D*^ with **P** ^*Q*^ [Eq. (4)], we recognize that the nonclassical polarization has the same mathematical form as the deformation polarization. In view of this, the nonclassical effects and the geometrical deformations can thus be treated under the same framework by defining a generalized *d*-parameter, *d*_eff_, with

(8) $${\bar{\bar{d}}}_{\text{eff}}\left(r\right)=h\left(r\right)\bar{\bar{I}}+\bar{\bar{d}}.$$

Apparently, this treatment can be applied for modeling statistical optical responses of particle ensembles that generally have tiny shape variations due to fabrication imperfections (Figure 4).

The above observation offers a geometrical interpretation to the Feibelman *d*-parameters. Particularly, when *d*_⊥_ = *d*_∥_, the nonclassical effects are equivalent to an isotropic geometrical deformation with *h* = *d*_⊥_. In general cases with *d*_⊥_ ≠ *d*_∥_, this strict equivalence breaks down. Nevertheless, the recognition of the unified treatment of the nonclassical effects and the geometrical deformation still offers an appealing perspective for better understanding the physical meaning of the Feibelman *d*-parameters.

## 3 Numerical validations

### 3.1 Single nanoparticles

We first study scattering of a plane wave by metal spheres. Two different metals, Ag (silver) and Al (aluminum), are considered. The diameters of the spheres are varied from 80 nm (with noticeable retardation effects) to 2 nm (where the nonclassical effects become important). The synthesis of metal nanoparticles widely uses colloid chemistry in liquid environments with, e.g. water as solvent [33, 36]. We thus here choose water as the embedding medium with a refractive index of 1.33. For Ag, the nonclassical effects are included by setting *d*_⊥_ = −0.5 + 0.3i nm. Specifically, the real part of *d*_⊥_ is determined by fitting the experimental data of the size-dependent resonance shift [20] to the perturbation theory established in Reference [32], while the imaginary part is chosen to reproduce the well-known Kreibig damping [37] *v*_F_/*R* (*v*_F_, Fermi velocity; *R*, sphere radius). For Al, we use *d*_⊥_ predicted with the time-dependent density-functional theory [38], which reasonably fits with the experimental observations [39]. The impact of *d*_∥_ is known to be negligible for charge-neutral materials, and we thus set *d*_∥_ = 0 [16]. Moreover, we note that *d*-parameters depend on specific dielectric environments surrounding metals. The used *d*_⊥_’s here omit this dependence [40], and are thus approximate. Nevertheless, the main purpose of the present work focusing on clarifying the validity of the modal method is unaffected by this approximation.

Figure 2(A) plots the extinction cross-section spectra of the spheres, comparing the predictions from the modal method and the full numerical results obtained with COMSOL Multiphysics [25, 41]. Note that, in the COMSOL simulations, the effects of the *d*-parameters are incorporated by introducing an auxiliary potential, see Reference [25] for details. We focus on the spectral region wherein the lowest-order dipolar mode is dominant (see the inset in Figure 2(A) for the modal profile). Therefore, only a single dipolar mode is used in the modal method, and the independent-mode approximation [Eq. (7a)] is employed to compute the modal coefficient. The comparisons validate the accuracy of the modal method. In Supplementary Figure S1, we further validate the modal method by comparing its results wiwith the analytic solutions predicted by the mesoscopic Mie’s theory that incorporates *d*-parameters [42]. Moreover, as shown in Supplementary Figure S2(A), the modal method continually to provide reliable predictions for spheres with diameter as large as 200–300 nm.

Notably, as the sphere diameter increases in the range (roughly) above 10 nm, the dipolar resonances show red shifts along with line broadening. This phenomenon is due to the retardation effects and is precisely reproduced by the modal method. On the other hand, as the sphere diameter decreases in the deep-nanometer scale below 10 nm, the resonance shows 1/*D* (*D*, diameter) size-dependent shifts and broadening. This is due to the nonclassical effects. Specifically, for Ag with Re(*d*_⊥_) < 0, the resonant blueshifts are observed [19, 20], while for Al with Re(*d*_⊥_) > 0, the opposite redshifts instead occur [21]. Moreover, the 1/*D*-dependent broadening is known as the Kreibig damping [37], which in our formalism is characterized by the imaginary part of *d*_⊥_.

To further validate the predictive accuracy of the modal method, we additionally test nanoparticles of other shapes. Figure 2(B) and Supplementary Figure S2(B) illustrate the extinction cross-section spectra for rod structures. The comparisons between the modal method and the full numerical solutions present quantitative agreements.

### 3.2 Multi-scale plasmonic architectures

Plasmonic dimers, film-coupled plasmonic nanoresonators and other similar structures, with particle dimensions *D* comparable to light wavelength and gap sizes *g* close to electronic length scales (Figure 1(B)), are well known for supporting extreme light–matter interactions inside tiny-gap regions [17, 23, 35]. In these multi-scale structures, the retardation and nonclassical effects coexist, interplay with plasmon oscillations, thereby significantly affecting optical responses. In this regard, their simulations require electrodynamic solvers that incorporate both the retardation and nonclassical effects, which just fits the application scope of the present modal method.

We first consider a spherical dimer. The dimer hosts strong hybridizations of plasmon resonances of individual spheres, leading to rich resonance features. To include multiple resonance features, we here retain the four lowest-order electrostatic SPRs in the modal method (see Supplementary Figure S3 for modal profiles). The dimer is made of Ag with spherical diameter 80 nm. We vary the gap distance between two spheres from 10 nm to 1 nm. Figure 3(A) compares the extinction cross-section spectra computed with the modal method with the numerical solutions obtained with COMSOL Multiphysics. Referring to the modal method, we note that the modal couplings due to the retardation and nonclassical effects cannot be neglected in this case (see Supplementary Figure S5). Hence, we compute the modal coefficients by solving the full matrix of Eq. (4), i.e., retaining the off-diagonal terms in the Hamiltonian, instead of using the simplified independent-mode approximation. The excellent agreements between the modal results and the fully numerical solutions prove the soundness of the modal method for describing optical responses of complex plasmonic structures. Besides, to highlight the significant impact of the nonclassical effects at small gap sizes, we also plot the spectra under the LRA by setting *d*_⊥_ = 0 for 1 nm and 2 nm gaps. The comparisons between the nonclassical and LRA results show that the nonclassical effects lead to considerable resonance shifts and damping broadening, which have been observed in recent experiments [23, 25]. Next, we consider a bow-tie structure. Figure 3(B) plots the extinction cross-section spectra. In the modal method, we use the four lowest-order static modes in the modal method (see Supplementary Figure S4 for modal profiles). The comparisons between the modal method and the full numerical solutions again show quantitative agreements.

### 3.3 Geometrically deformed nanoparticles

As discussed above, optical responses of a set of nanoparticles, deformed from an original nanoparticle, can be conveniently treated (to the leading order of the deformation) by defining a generalized *d*-parameter, ${\bar{\bar{d}}}_{\text{eff}}$ , by simply adding the deformation contribution on top of the nonclassical *d*-parameter [see Eq. (8) and Figure 4(A)]. To validate this idea, we consider a sphere and take its electrostatic plasmon modes to predict the extinction cross-section spectra of spheroids. A single dipolar mode (polarized in the direction of the incident electric field) is retained in the modal formalism. In Figure 4(B), we compare the spectra predicted with the modal method to the full numerical solutions obtained with COMSOL Multiphysics. The qualitative agreements between two approaches validate the effectiveness of the modal method for predicting optical responses of geometrically deformed particles. Note that, the numerical results show two spectral peaks, while the modal method only predicts the long-wavelength one due to the use of a single dipolar mode. Moreover, since ${\bar{\bar{d}}}_{\text{eff}}$ includes both perpendicular and parallel components (with respect to the particle boundary ∂Ω), the above results also validate that the present method can handle general cases with both nonzero *d*_⊥_ and *d*_∥_ equally well.

## 4 Conclusions

In the present paper, we develop an analytic approach that uses electrostatic surface plasmon modes as the basis to model optical responses of metal nanoparticles. The retardation effects of light and nonclassical effects of electrons are treated on an equal footing. The developed approach saves computational time (see discussions in Supplementary Section S3) and offers physical intuitions. Its predictive accuracy is validated for single nanoparticles of different morphologies, complex multi-scale structures (such as dimer structures), and deformed particles, with feature sizes from hundreds of nanometers to a few nanometers. We envision that the proposed method could be useful tool for optimizing and designing plasmonic nanoresonators [43].

## Supplementary Material

Supplementary Material Details
