# Supplementary material for: Efficient modal analysis of plasmonic nanoparticles: from retardation to nonclassical regimes
Source: Nanophotonics. 2022 Feb 2;11(9):1887–95. doi: 10.1515/nanoph-2021-0668 (PMC11502095; doi:10.1515/nanoph-2021-0668)
Supplement: Supplementary file 1 — Supplementary Material Details [file j_nanoph-2021-0668_suppl.pdf]

SUPPLEMENTARY MATERIAL

Efficient modal analysis of plasmonic nanoparticles: from retardation to nonclassical regimes

Wei Yan[1,2,*] and Min Qiu[1,2,†]
[1]Key Laboratory of 3D Micro/Nano Fabrication and Characterization of Zhejiang Province, School of Engineering, Westlake University, 18 Shilongshan Road, Hangzhou 310024, Zhejiang Province, China
[2]Institute of Advanced Technology, Westlake Institute for Advanced Study, 18 Shilongshan Road, Hangzhou 310024, Zhejiang Province, China

CONTENTS

S1. Retarded-Nonclassical Surface Integral Formulation 1

S2. Modal Expansion 4

S3. Computation Speed 6

S4. Supplementary Figures 8

References 13

S1. RETARDED-NONCLASSICAL SURFACE INTEGRAL FORMULATION

In the electrostatic limit, surface plasmon resonances and their excitations by external incident fields are known to be well described by a classical surface integral formulation in terms of a surface charge density $\sigma(\mathbf{r})$ over a boundary domain $\mathbf{r} \in \partial\Omega$ separating a metal domain with its outer background. Below, we generalize the classical surface integral formulation by incorporating both retardation effects of light and nonclassical effects of electrons.

We start with Maxwell's wave equation for scattered electric field $\mathbf{E}_{\rm sca}$,

$$\left[\boldsymbol{\nabla}\times\boldsymbol{\nabla}\times-\omega^2\mu_0\varepsilon_{\rm bg}\right]\mathbf{E}_{\rm sca}(\mathbf{r}) = \omega^2\mu_0\Delta\varepsilon(\omega;\mathbf{r})\mathbf{E}_{\rm tot}(\mathbf{r}) + \omega^2\mu_0\mathbf{P}^{Q}(\omega;\mathbf{r}). \tag{S.1.1}$$

Here $\varepsilon_{\rm bg}$ denotes the background permittivity and $\Delta\varepsilon \equiv \varepsilon_{\rm np}-\varepsilon_{\rm bg}$ is the difference between the permittivities of the metal nanoparticle and the background; $\mathbf{P}^{Q}$ is the nonclassical surface polarization density placed on the background side of the metal boundary $\partial\Omega$, see Eq. (2) in the main text for its expression; $\hat{\mathbf{n}}$ denotes the unit normal at $\partial\Omega$ pointing toward the background; $\mathbf{E}_{\rm tot}$ is the total field, that is, the summation of the scattered field $\mathbf{E}_{\rm sca}$ and the incident electric field $\mathbf{E}_{\rm in}$.

We then introduce the background Green's tensor $\boldsymbol{G}_{\rm bg}$ satisfying

$$\left[\boldsymbol{\nabla}\times\boldsymbol{\nabla}-\omega^2\mu_0\varepsilon_{\rm bg}\right]\boldsymbol{G}_{\rm bg}(\omega;\mathbf{r},\mathbf{r}') = \bar{\bar{\boldsymbol{I}}}\delta(\mathbf{r}-\mathbf{r}'). \tag{S.1.2}$$

$\boldsymbol{G}_{\rm bg}$ is given by

$$\boldsymbol{G}_{\rm bg}(\omega;\mathbf{r},\mathbf{r}') = \left[\bar{\bar{\boldsymbol{I}}} + \frac{1}{k_{\rm bg}^2}\boldsymbol{\nabla}\boldsymbol{\nabla}\right]g_{\rm bg}(\omega;\mathbf{r},\mathbf{r}'), \tag{S.1.3}$$

with

$$g_{\mathrm{bg}}(\omega;\mathbf{r},\mathbf{r}') = \frac{\exp\left(ik_{\mathrm{bg}}|\mathbf{r}-\mathbf{r}'|\right)}{4\pi|\mathbf{r}-\mathbf{r}'|}, \tag{S.1.4}$$

and $k_{\mathrm{bg}} = \omega\sqrt{\varepsilon_{\mathrm{bg}}\mu_0}$.

$\mathbf{E}_{\mathrm{sca}}$ can be formulated in terms of $\boldsymbol{G}_{\mathrm{bg}}$ with

$$\begin{aligned}\mathbf{E}_{\mathrm{sca}}(\omega;\mathbf{r}) = \omega^2\mu_0\int_\Omega \boldsymbol{G}_{\mathrm{bg}}(\omega;\mathbf{r},\mathbf{r}')\cdot\Delta\varepsilon(\omega;\mathbf{r}')\mathbf{E}_{\mathrm{tot}}(\mathbf{r}')d^3\mathbf{r}' + \\ \lim_{\delta\to0^+}\omega^2\mu_0\oint_{\partial\Omega}\boldsymbol{G}_{\mathrm{bg}}(\omega;\mathbf{r},\mathbf{r}'+\delta\hat{\mathbf{n}})\cdot\mathbf{P}^Q(\mathbf{r}')d^2\mathbf{r}',\end{aligned} \tag{S.1.5}$$

where the volume and surface integral terms are performed over the domain and surface of the nanoparticle, respectively.

For convenience of analysis below, we partition $\mathbf{E}_{\mathrm{sca}}$ into the classical (cl) and nonclassical (ncl) parts:

$$\mathbf{E}_{\mathrm{sca}}(\mathbf{r}) = \mathbf{E}^{\mathrm{cl}}_{\mathrm{sca}}(\mathbf{r}) + \mathbf{E}^{\mathrm{ncl}}_{\mathrm{sca}}(\mathbf{r}), \tag{S.1.6}$$

with

$$\mathbf{E}^{\mathrm{cl}}_{\mathrm{sca}}(\mathbf{r}) = \omega^2\mu_0\int_\Omega \boldsymbol{G}_{\mathrm{bg}}(\omega;\mathbf{r},\mathbf{r}')\cdot\Delta\varepsilon(\omega;\mathbf{r}')\mathbf{E}_{\mathrm{tot}}(\mathbf{r}')d^3\mathbf{r}', \tag{S.1.7}$$

$$\mathbf{E}^{\mathrm{ncl}}_{\mathrm{sca}}(\mathbf{r}) = \lim_{\delta\to0^+}\omega^2\mu_0\oint_{\partial\Omega}\boldsymbol{G}_{\mathrm{bg}}(\omega;\mathbf{r},\mathbf{r}'+\delta\hat{\mathbf{n}})\cdot\mathbf{P}^Q(\mathbf{r}')d^2\mathbf{r}'. \tag{S.1.8}$$

$\mathbf{E}^{\mathrm{cl}}_{\mathrm{sca}}$ in the domain of the nanoparticle ($\mathbf{r}\in\partial\Omega$) can be transformed to a surface integral with the following manipulations:

$$\begin{aligned}\mathbf{E}^{\mathrm{cl}}_{\mathrm{sca}}(\mathbf{r}) &= \omega^2\mu_0\int_\Omega \boldsymbol{G}_{\mathrm{bg}}(\omega;\mathbf{r},\mathbf{r}')\cdot\Delta\varepsilon(\omega)\mathbf{E}_{\mathrm{tot}}(\mathbf{r}')d^3\mathbf{r}' \\ &\overset{a}{=} \frac{1}{\varepsilon_{\mathrm{bg}}}\int_\Omega\left[\boldsymbol{\nabla}'\times\boldsymbol{\nabla}'\times\boldsymbol{G}_{\mathrm{bg}}(\omega;\mathbf{r},\mathbf{r}')\right]\cdot\Delta\varepsilon(\omega)\mathbf{E}_{\mathrm{tot}}(\mathbf{r}')d^3\mathbf{r}' - \frac{\Delta\varepsilon(\omega)}{\varepsilon_{\mathrm{bg}}}\mathbf{E}_{\mathrm{tot}}(\mathbf{r}) \\ &\overset{b}{=} \frac{1}{\varepsilon_{\mathrm{bg}}}\int_\Omega\boldsymbol{\nabla}'\cdot\left[\left(\boldsymbol{\nabla}'\times\boldsymbol{G}_{\mathrm{bg}}(\omega;\mathbf{r},\mathbf{r}')\right)\times\Delta\varepsilon(\omega)\mathbf{E}_{\mathrm{tot}}(\mathbf{r}')d^3\mathbf{r}'\right] + \\ &\quad \frac{1}{\varepsilon_{\mathrm{bg}}}\int_\Omega\left[\boldsymbol{\nabla}'\times\boldsymbol{G}_{\mathrm{bg}}(\omega;\mathbf{r},\mathbf{r}')\right]\cdot\left[\boldsymbol{\nabla}'\times\Delta\varepsilon(\omega)\mathbf{E}_{\mathrm{tot}}(\mathbf{r}')\right]d^3\mathbf{r}' - \frac{\Delta\varepsilon(\omega)}{\varepsilon_{\mathrm{bg}}}\mathbf{E}_{\mathrm{tot}}(\mathbf{r}) \\ &\overset{c}{\simeq} \frac{1}{\varepsilon_{\mathrm{bg}}}\int_\Omega\boldsymbol{\nabla}'\cdot\left[\left(\boldsymbol{\nabla}'\times\boldsymbol{G}_{\mathrm{bg}}(\omega;\mathbf{r},\mathbf{r}')\right)\times\Delta\varepsilon(\omega)\mathbf{E}_{\mathrm{tot}}(\mathbf{r}')\right]d^3\mathbf{r}' - \frac{\Delta\varepsilon(\omega)}{\varepsilon_{\mathrm{bg}}}\mathbf{E}_{\mathrm{tot}}(\mathbf{r}) \\ &\overset{d}{=} \frac{1}{\varepsilon_{\mathrm{bg}}}\oint_{\partial\Omega}\boldsymbol{\nabla}'g_{\mathrm{bg}}(\omega;\mathbf{r},\mathbf{r}')\times\left[\hat{\mathbf{n}}(\mathbf{r}')\times\Delta\varepsilon(\omega)\mathbf{E}_{\mathrm{tot}}(\mathbf{r}')\right]d^2\mathbf{r}' - \frac{\Delta\varepsilon(\omega)}{\varepsilon_{\mathrm{bg}}}\mathbf{E}_{\mathrm{tot}}(\mathbf{r}) \\ &\overset{e}{=} \lim_{\delta\to0^+}\frac{1}{\varepsilon_{\mathrm{bg}}}\oint_{\partial\Omega}\boldsymbol{\nabla}'g^{\mathrm{static}}_{\mathrm{bg}}(\mathbf{r},\mathbf{r}')\cdot\left[\hat{\mathbf{n}}(\mathbf{r}')\cdot\Delta\varepsilon(\omega)\mathbf{E}_{\mathrm{tot}}[\mathbf{r}'-\delta\hat{\mathbf{n}}(\mathbf{r}')]\right]d^2\mathbf{r}' + \\ &\quad \frac{1}{\varepsilon_{\mathrm{bg}}}\oint_{\partial\Omega}\boldsymbol{\nabla}'g^{\mathrm{ret}}_{\mathrm{bg}}(\omega;\mathbf{r},\mathbf{r}')\times\left[\hat{\mathbf{n}}(\mathbf{r}')\times\Delta\varepsilon(\omega;\mathbf{r}')\mathbf{E}_{\mathrm{tot}}(\mathbf{r}')\right]d^2\mathbf{r}'.\end{aligned} \tag{S.1.9}$$

The following steps are used in the above derivations

a. Application of Eq. (S.1.2), i.e., $\boldsymbol{G}_{\mathrm{bg}}(\omega;\mathbf{r},\mathbf{r}') = \frac{1}{\omega^2\mu_0\varepsilon_{\mathrm{bg}}}\boldsymbol{\nabla}\times\boldsymbol{\nabla}\boldsymbol{G}_{\mathrm{bg}}(\omega;\mathbf{r},\mathbf{r}') - \frac{1}{\omega^2\mu_0\varepsilon_{\mathrm{bg}}}\bar{\bar{\boldsymbol{I}}}\delta(\mathbf{r}-\mathbf{r}')$ and note that $\mathbf{r}\in\Omega$.

b. Application of the vector identity $\boldsymbol{\nabla}'\cdot(\mathbf{a}\times\mathbf{b}) = \mathbf{b}\cdot\boldsymbol{\nabla}'\times\mathbf{a} - \mathbf{a}\cdot\boldsymbol{\nabla}'\times\mathbf{b}$ with $\mathbf{a} = \boldsymbol{\nabla}'\times\boldsymbol{G}_{\mathrm{bg}}(\omega;\mathbf{r},\mathbf{r}')$ and $\mathbf{b} = \Delta\varepsilon(\omega)\mathbf{E}_{\mathrm{tot}}(\mathbf{r}')$; note that the gradient operator $\boldsymbol{\nabla}'$ is applied to the variable $\mathbf{r}'$.

c. Employment of the electrostatic approximation $\boldsymbol{\nabla}\times\mathbf{E}_{\mathrm{tot}}(\mathbf{r})\simeq 0$.

d. Application of Eq. (S.1.3) and using the divergence theorem to transform the volume integral to the surface integral.

e. First, decomposing g_{bg} into

$$g_{\text{bg}} = g_{\text{bg}}^{\text{static}} + g_{\text{bg}}^{\text{ret}}, \tag{S.1.10}$$

with

$$g_{\text{bg}}^{\text{static}}(\mathbf{r},\mathbf{r}') = \frac{1}{4\pi|\mathbf{r}-\mathbf{r}'|}, \quad g_{\text{bg}}^{\text{ret}}(\omega;\mathbf{r},\mathbf{r}') = \frac{\exp\left(ik_{\text{bg}}|\mathbf{r}-\mathbf{r}'|\right)-1}{4\pi|\mathbf{r}-\mathbf{r}'|}, \tag{S.1.11}$$

where$g_{\text{bg}}^{\text{static}}$ is the static scalar Green's function, while $g_{\text{bg}}^{\text{ret}}$ accounts for the retardation effects. Then, performing the following manipulations,

$$\begin{aligned}
&\frac{1}{\varepsilon_{\text{bg}}}\oint_{\partial\Omega} \boldsymbol{\nabla}' g_{\text{bg}}^{\text{static}}(\mathbf{r},\mathbf{r}') \times [\hat{\mathbf{n}}(\mathbf{r}') \times \Delta\varepsilon(\omega;\mathbf{r}')\mathbf{E}_{\text{tot}}(\mathbf{r}')]\, d^2\mathbf{r}' \\
&= \frac{1}{\varepsilon_{\text{bg}}}\oint_{\partial\Omega} \left[\boldsymbol{\nabla}' g_{\text{bg}}^{\text{static}}(\mathbf{r},\mathbf{r}') \cdot \Delta\varepsilon(\omega)\mathbf{E}_{\text{tot}}(\mathbf{r}')\right]\hat{\mathbf{n}}(\mathbf{r}') - \\
&\qquad \left[\boldsymbol{\nabla}' g_{\text{bg}}^{\text{static}}(\mathbf{r},\mathbf{r}') \cdot \hat{\mathbf{n}}(\mathbf{r}')\right]\Delta\varepsilon(\omega)\mathbf{E}_{\text{tot}}(\mathbf{r}') d^2\mathbf{r}' \\
&= \frac{1}{\varepsilon_{\text{bg}}}\int_{\Omega} \boldsymbol{\nabla}'\left[\boldsymbol{\nabla}' g_{\text{bg}}^{\text{static}}(\mathbf{r},\mathbf{r}') \cdot \Delta\varepsilon(\omega;\mathbf{r}')\mathbf{E}_{\text{tot}}(\mathbf{r}')\right] - \boldsymbol{\nabla}' \cdot \left[\boldsymbol{\nabla}' g_{\text{bg}}^{\text{static}}(\mathbf{r},\mathbf{r}')\mathbf{E}_{\text{tot}}(\mathbf{r}')\right] d^3\mathbf{r}' \\
&= \frac{1}{\varepsilon_{\text{bg}}}\int_{\Omega} \Delta\varepsilon(\omega)\mathbf{E}_{\text{tot}}(\mathbf{r}') \cdot \boldsymbol{\nabla}'\boldsymbol{\nabla}' g_{\text{bg}}^{\text{static}}(\mathbf{r},\mathbf{r}') - \Delta\varepsilon(\omega)\mathbf{E}_{\text{tot}}(\mathbf{r}')\boldsymbol{\nabla}'^2 g_{\text{bg}}^{\text{static}}(\mathbf{r},\mathbf{r}') d^3\mathbf{r}' \\
&= \frac{1}{\varepsilon_{\text{bg}}}\int_{\Omega} \boldsymbol{\nabla}' \cdot \left[\Delta\varepsilon(\omega)\mathbf{E}_{\text{tot}}(\mathbf{r}')\boldsymbol{\nabla}' g_{\text{bg}}^{\text{static}}(\mathbf{r},\mathbf{r}')\right] d^3\mathbf{r}' + \frac{\Delta\varepsilon(\omega)}{\varepsilon_{\text{bg}}}\mathbf{E}_{\text{tot}}(\mathbf{r}) \\
&= \lim_{\delta\to 0^+}\frac{1}{\varepsilon_{\text{bg}}}\oint_{\partial\Omega} \boldsymbol{\nabla}' g_{\text{bg}}^{\text{static}}(\mathbf{r},\mathbf{r}')\left[\hat{\mathbf{n}}(\mathbf{r}') \cdot \Delta\varepsilon(\omega)\mathbf{E}_{\text{tot}}[\mathbf{r}' - \delta\hat{\mathbf{n}}(\mathbf{r}')]\right] d^2\mathbf{r}' + \frac{\Delta\varepsilon(\omega)}{\varepsilon_{\text{bg}}}\mathbf{E}_{\text{tot}}(\mathbf{r}').
\end{aligned} \tag{S.1.12}$$

Inserting Eq. (S.1.9) into Eq. (S.1.6), we obtain that

$$\begin{aligned}
\mathbf{E}_{\text{sca}}(\mathbf{r}) = {} & \lim_{\delta\to 0^+}\frac{1}{\varepsilon_{\text{bg}}}\oint_{\partial\Omega} \boldsymbol{\nabla}' g_{\text{bg}}^{\text{static}}(\mathbf{r},\mathbf{r}') \cdot [\hat{\mathbf{n}}(\mathbf{r}') \cdot \Delta\varepsilon(\omega;\mathbf{r}')\mathbf{E}_{\text{tot}}[\mathbf{r}' - \hat{\mathbf{n}}(\mathbf{r}')]]\, d^2\mathbf{r}' + \\
& \frac{1}{\varepsilon_{\text{bg}}}\oint_{\partial\Omega} \boldsymbol{\nabla}' g_{\text{bg}}^{\text{ret}}(\omega;\mathbf{r},\mathbf{r}') \times [\hat{\mathbf{n}}(\mathbf{r}') \times \Delta\varepsilon(\omega;\mathbf{r}')\mathbf{E}_{\text{tot}}(\mathbf{r}')]\, d^2\mathbf{r}' + \\
& \lim_{\delta\to 0^+}\omega^2\mu_0\oint_{\partial\Omega} \boldsymbol{G}_{\text{bg}}(\omega;\mathbf{r},\mathbf{r}' + \delta\hat{\mathbf{n}}) \cdot \mathbf{P}^{Q}(\mathbf{r}') d^2\mathbf{r}'.
\end{aligned} \tag{S.1.13}$$

To cast Eq. (S.1.13) into a form resembling the well known electrostatic surface integral formalism, we define the surface charge density on the $\partial\Omega$

$$\sigma_{\text{tot}}(\mathbf{r}) \equiv \lim_{\delta\to 0^+}\frac{\Delta\varepsilon(\omega)}{\varepsilon_{\text{bg}}}\left[\hat{\mathbf{n}}(\mathbf{r}) \cdot \mathbf{E}_{\text{tot}}(\mathbf{r} - \delta\hat{\mathbf{n}})\right]. \tag{S.1.14}$$

We then reformulate Eq. (S.1.13) into

$$\begin{aligned}
\frac{\varepsilon_{\text{bg}}}{\Delta\varepsilon}\sigma_{\text{tot}}(\mathbf{r}) = {} & \oint_{\partial\Omega} \hat{\mathbf{n}}(\mathbf{r}) \cdot \boldsymbol{\nabla}' g_{\text{bg}}^{\text{static}}(\mathbf{r},\mathbf{r}') \cdot \sigma_{\text{tot}}(\mathbf{r}') d^2\mathbf{r}' + \\
& \oint_{\partial\Omega} \hat{\mathbf{n}}(\mathbf{r}) \cdot \boldsymbol{\nabla}' g_{\text{bg}}^{\text{ret}}(\omega;\mathbf{r},\mathbf{r}') \times \left[\hat{\mathbf{n}}(\mathbf{r}') \times \frac{\Delta\varepsilon(\omega)}{\varepsilon_{\text{bg}}}\mathbf{E}_{\text{tot}}(\mathbf{r}')\right] d^2\mathbf{r}' + \\
& \lim_{\delta\to 0^+}\omega^2\mu_0\oint_{\partial\Omega} \hat{\mathbf{n}}(\mathbf{r}) \cdot \boldsymbol{G}_{\text{bg}}(\omega;\mathbf{r},\mathbf{r}' + \delta\hat{\mathbf{n}}) \cdot \mathbf{P}^{Q}(\mathbf{r}') d^2\mathbf{r}' + \\
& \hat{\mathbf{n}}(\mathbf{r}) \cdot \mathbf{E}_{\text{in}}(\mathbf{r}).
\end{aligned} \tag{S.1.15}$$

Equation (S.1.15) is the derived surface integral formalism that incorporates both the retardation and nonclassical effects.

S2. MODAL EXPANSION

We solve Eq. (S.1.15) using the basis of the electrostatic plasmon modes, which are eigensolutions of the classical electrostatic surface integral equation [i.e., the first line of Eq. (S.1.15)],

$$\Lambda_m \sigma_m(\mathbf{r}) = \oint_{\partial\Omega} \hat{\mathbf{n}}(\mathbf{r}) \cdot \nabla' g_{\text{bg}}^{\text{static}}(\mathbf{r}, \mathbf{r}') \cdot \sigma_m(\mathbf{r}) d^2\mathbf{r}'. \tag{S.2.1}$$

where $\{\Lambda_1, \Lambda_2, \Lambda_3, \cdots\}$ denote eigenvalues. The biorthogonal partner of eigensolution σ_m (charge) is ϕ_m (potential) given by

$$\phi_m(\mathbf{r}) \equiv \oint_{\partial\Omega} g_{\text{bg}}^{\text{static}}(\mathbf{r}, \mathbf{r}') \sigma_m(\mathbf{r}') d^2\mathbf{r}'. \tag{S.2.2}$$

The orthonormal condition of the modes is defined as

$$\oint_{\partial\Omega} \phi_m(\mathbf{r}) \sigma_n(\mathbf{r}) d^2\mathbf{r} = L^3 \delta_{nm}, \tag{S.2.3}$$

where $\delta_{nm} = 1$ for $n = m$ and 0 otherwise; L is a characteristic length of the nanoparticle that can be set arbitrarily.

We make an ansatz expanding the unknowns in Eq. (S.1.15) with the basis of the electrostatic modes:

$$\sigma_{\text{tot}}(\mathbf{r}) = \hat{\mathbf{n}}(\mathbf{r}) \cdot \frac{\Delta\varepsilon(\omega)}{\varepsilon_{\text{bg}}} \mathbf{E}_{\text{tot}}(\mathbf{r}) = \sum_m \alpha_m \sigma_m(\mathbf{r}), \tag{S.2.4a}$$

$$\Delta\varepsilon(\omega) \mathbf{E}_{\text{tot}}(\mathbf{r}) = -\sum_m \alpha_m \Delta\varepsilon_m \nabla\phi_m(\mathbf{r}), \tag{S.2.4b}$$

where α_m denotes the expansion coefficient.

Then, we insert Eqs. (S.2.4) into Eq. (S.1.15), and apply $\oint_{\partial\Omega} d^2\mathbf{r}\phi_n(\mathbf{r})$ to both sides of the equation. Finally, we arrive at that

$$\Lambda(\omega)\alpha_n = \mathcal{H}_{nm}^0 \alpha_m + \mathcal{H}_{nm}^R \alpha_m + \mathcal{H}_{nm}^Q \alpha_m + +\mathcal{H}_{nm}^{R-Q} \alpha_m, \tag{S.2.5}$$

with the expressions of $\mathcal{H}^{0,R,Q,R-Q}$ given in Eqs.(5)-(6) in the main text.

The derivations of $\mathcal{H}^R$ are given as follows:

$$\begin{aligned}
\mathcal{H}_{nm}^R &= \frac{1}{\Lambda_m L^3} \oint_{\partial\Omega} \oint_{\partial\Omega} \phi_n(\mathbf{r}) \hat{\mathbf{n}}(\mathbf{r}) \cdot \nabla' g_{\text{bg}}^{\text{ret}}(\omega; \mathbf{r}, \mathbf{r}') \times [\hat{\mathbf{n}}(\mathbf{r}') \times \mathbf{E}_m(\mathbf{r}')]\, d^2\mathbf{r} d^2\mathbf{r}' \\
&= \frac{1}{\Lambda_m L^3} \oint_{\partial\Omega} \oint_{\partial\Omega} g_{\text{bg}}^{\text{ret}}(\omega; \mathbf{r}, \mathbf{r}')\, [\hat{\mathbf{n}}(\mathbf{r}) \times \nabla\phi_n(\mathbf{r})] \cdot [\hat{\mathbf{n}}(\mathbf{r}') \times \mathbf{E}_m(\mathbf{r}')]\, d^2\mathbf{r} d^2\mathbf{r}' \\
&\overset{a}{=} -\sum_{k=1,2,\cdots} \frac{i^k (s^R)^k}{4\pi \Lambda_m L^{k+3}} \oint_{\partial\Omega} \oint_{\partial\Omega} |\mathbf{r} - \mathbf{r}'|^{k-1}\, [\hat{\mathbf{n}}(\mathbf{r}) \times \mathbf{E}_n(\mathbf{r})] \cdot [\hat{\mathbf{n}}(\mathbf{r}') \times \mathbf{E}_m(\mathbf{r}')]\, d^2\mathbf{r} d^2\mathbf{r}' \\
&\overset{b}{=} -\sum_{k=2,3,\cdots} \frac{i^k (s^R)^k}{4\pi \Lambda_m \Lambda_m L^{k+3}} \oint_{\partial\Omega} \oint_{\partial\Omega} |\mathbf{r} - \mathbf{r}'|^{k-1}\, [\hat{\mathbf{n}}(\mathbf{r}) \times \mathbf{E}_n(\mathbf{r})] \cdot [\hat{\mathbf{n}}(\mathbf{r}') \times \mathbf{E}_m(\mathbf{r}')]\, d^2\mathbf{r} d^2\mathbf{r}' \\
&\overset{c}{\equiv} \sum_{k=2,3,\cdots} f_{nm;k}^R (s^R)^k,
\end{aligned} \tag{S.2.6}$$

where the following steps are used

a. Application of Taylor series expansion of $g^{\text{ret}}_{\text{bg}}(\omega;\mathbf{r},\mathbf{r}')$,

$$g^{\text{ret}}_{\text{bg}}(\omega;\mathbf{r},\mathbf{r}') = \sum_{k=1,2,\cdots} \frac{i^k|\mathbf{r}-\mathbf{r}'|^{k-1}k^k_{\text{bg}}}{4\pi}.$$

b. The term $k = 1$, $\oint_{\partial\Omega}\oint_{\partial\Omega}[\hat{\mathbf{n}}(\mathbf{r})\times\mathbf{E}_n(\mathbf{r})]\cdot[\hat{\mathbf{n}}(\mathbf{r}')\times\mathbf{E}_m(\mathbf{r}')]\,d^2\mathbf{r}d^2\mathbf{r}'$, equals zero, which can be derived with

$$\oint_{\partial\Omega}\hat{\mathbf{n}}(\mathbf{r})\times\mathbf{E}_{n,m}(\mathbf{r})d^2\mathbf{r} = \int_{\Omega}\nabla\times\mathbf{E}_{n,m}(\mathbf{r})d^3\mathbf{r} = 0.$$

c. Application of the definitions

$$s^R \equiv k_{\text{bg}}L,$$
$$f^R_{nm;k} \equiv -\frac{i^k}{4\pi\Lambda_m L^{k+3}}\oint_{\partial\Omega}\oint_{\partial\Omega}|\mathbf{r}-\mathbf{r}'|^{k-1}\,[\hat{\mathbf{n}}(\mathbf{r})\times\mathbf{E}_m(\mathbf{r})]\,.$$

The detailed derivations for $\mathcal{H}^{Q,R-Q}$ are given by

$$\begin{aligned}
\mathcal{H}^{Q}_{nm}+\mathcal{H}^{R-Q}_{nm} &\overset{a}{=} \lim_{\delta\to0^+}\omega^2\mu_0\frac{\Delta\varepsilon_m}{L^3}\oint_{\partial\Omega}\oint_{\partial\Omega}\phi_n(\mathbf{r})\hat{\mathbf{n}}(\mathbf{r})\cdot\boldsymbol{G}_{\text{bg}}(\omega;\mathbf{r},\mathbf{r}'+\delta\hat{\mathbf{n}})\cdot\bar{\bar{\mathbf{d}}}(\omega)\cdot\mathbf{E}_m(\mathbf{r}'-\delta\hat{\mathbf{n}}')d^2\mathbf{r}'d^2\mathbf{r}\\
&\overset{b}{=} -\lim_{\delta\to0^+}\omega^2\mu_0\frac{\Delta\varepsilon_m}{L^3}\int_{\Omega}\oint_{\partial\Omega}\mathbf{E}_n(\mathbf{r})\cdot\boldsymbol{G}_{\text{bg}}(\omega;\mathbf{r},\mathbf{r}'+\delta\hat{\mathbf{n}})\cdot\bar{\bar{\mathbf{d}}}\cdot\mathbf{E}_m(\mathbf{r}'-\delta\hat{\mathbf{n}}')d^2\mathbf{r}'d^3\mathbf{r}\\
&\overset{c}{=} \underbrace{-\lim_{\delta\to0^+}\frac{\Delta\varepsilon_m}{\varepsilon_{\text{bg}}L^3}\oint_{\partial\Omega}\oint_{\partial\Omega}[\hat{\mathbf{n}}(\mathbf{r})\cdot\mathbf{E}_n(\mathbf{r}-\delta\hat{\mathbf{n}})]\,\nabla g^{\text{static}}_{\text{bg}}(\mathbf{r},\mathbf{r}'+\delta\hat{\mathbf{n}}')\cdot\bar{\bar{\mathbf{d}}}(\omega)\cdot\mathbf{E}_m(\mathbf{r}'-\delta\hat{\mathbf{n}}')d^2\mathbf{r}'d^2\mathbf{r}}_{\equiv\mathcal{H}^{Q}_{nm}}\\
&\quad\underbrace{-\lim_{\delta\to0^+}\frac{\Delta\varepsilon_m}{\varepsilon_{\text{bg}}L^3}\oint_{\partial\Omega}\oint_{\partial\Omega}\left\{\nabla g^{\text{ret}}_{\text{bg}}(\omega;\mathbf{r},\mathbf{r}')\times[\hat{\mathbf{n}}(\mathbf{r})\times\mathbf{E}_n(\mathbf{r}-\delta\hat{\mathbf{n}})]\right\}\cdot\bar{\bar{\mathbf{d}}}(\omega)\cdot\mathbf{E}_m(\mathbf{r}'-\delta\hat{\mathbf{n}}')d^2\mathbf{r}'d^2\mathbf{r}}_{\equiv\mathcal{H}^{R-Q}_{nm}},
\end{aligned} \tag{S.2.7}$$

where the following steps are used

a. Employment of the definition of $\mathbf{P}^Q$ introduced in the main text

$$\mathbf{P}^Q(\omega;\mathbf{r}) \equiv \lim_{\delta\to0^+}\Delta\varepsilon(\omega)\bar{\bar{\mathbf{d}}}(\omega)\cdot\mathbf{E}_{\text{tot}}(\mathbf{r}-\delta\hat{\mathbf{n}}),$$

and noting that $\mathbf{P}^Q$ is placed on the background side of the particle boundary $\partial\Omega$. Application of the modal expansion for $\Delta\varepsilon(\omega)\mathbf{E}_{\text{tot}}(\mathbf{r}-\delta\hat{\mathbf{n}})$, see Eq. (S.2.4).

b. Transforming the surface integral $\oint_{\partial\Omega}\phi_n(\mathbf{r})\hat{\mathbf{n}}\cdot\boldsymbol{G}_{\text{bg}}(\omega;\mathbf{r},\mathbf{r}'+\delta\hat{\mathbf{n}})d^2\mathbf{r}$ to the volume integral by using the divergence theorem.

c. Repeating the derivations detailed in Eq. (S.1.9).

In the above, by partitioning the static and retardation terms in Green's function, we have explicitly

expressed $\mathcal{H}_{nm}^{Q}$ and $\mathcal{H}_{nm}^{R-Q}$, respectively. Below, we further simplify the expression of $\mathcal{H}_{nm}^{Q,R}$.

$$\begin{aligned}\mathcal{H}_{nm}^{Q} &= -\lim_{\delta\to 0^+}\frac{\Delta\varepsilon_m}{\varepsilon_{\text{bg}}L^3}\oint_{\partial\Omega}\oint_{\partial\Omega}[\hat{\mathbf{n}}(\mathbf{r})\cdot\mathbf{E}_n(\mathbf{r}-\delta\hat{\mathbf{n}})]\,\nabla g_{\text{bg}}^{\text{static}}(\mathbf{r},\mathbf{r}'+\delta\hat{\mathbf{n}}')\cdot\overline{\overline{\mathbf{d}}}(\omega)\cdot\mathbf{E}_m(\mathbf{r}'-\delta\hat{\mathbf{n}}')d^2\mathbf{r}'d^2\mathbf{r}\\ &= -\lim_{\delta\to 0^+}\frac{\Delta\varepsilon_m}{\Delta\varepsilon_n L^3}\oint_{\partial\Omega}\oint_{\partial\Omega}\sigma_n(\mathbf{r})\nabla g_{\text{bg}}^{\text{static}}(\mathbf{r},\mathbf{r}'+\delta\hat{\mathbf{n}}')\cdot\overline{\overline{\mathbf{d}}}(\omega)\cdot\mathbf{E}_m(\mathbf{r}'-\delta\hat{\mathbf{n}}')d^2\mathbf{r}'d^2\mathbf{r}\\ &\overset{a}{=} -\lim_{\delta\to 0^+}\frac{\Delta\varepsilon_m}{\Delta\varepsilon_n L^3}\oint_{\partial\Omega}\mathbf{E}_n(\mathbf{r}'+\delta\hat{\mathbf{n}}')\cdot\overline{\overline{\mathbf{d}}}(\omega)\cdot\mathbf{E}_m(\mathbf{r}'-\delta\hat{\mathbf{n}}')\\ &\overset{b}{=} \overline{\overline{\mathbf{f}}}_{nm}^{Q} : \mathbf{s}^{Q},\end{aligned} \tag{S.2.8}$$

where the following steps are used

a.

$$\begin{aligned}\oint\sigma_n(\mathbf{r})\nabla g_{\text{bg}}^{\text{static}}(\mathbf{r},\mathbf{r}'+\delta\hat{\mathbf{n}}')d^2\mathbf{r} &= -\nabla'\oint\sigma_n(\mathbf{r})g_{\text{bg}}^{\text{static}}(\mathbf{r},\mathbf{r}'+\delta\hat{\mathbf{n}}')d^2\mathbf{r}\\ &= -\nabla'\phi_n(\mathbf{r}'+\delta\hat{\mathbf{n}}') = \mathbf{E}_n(\mathbf{r}'+\delta\hat{\mathbf{n}}').\end{aligned}$$

b. Application of the definitions of $\overline{\overline{\mathbf{f}}}_{nm}^{Q}$ and $\mathbf{s}^{Q}$:

$$\mathbf{s}^{Q} \equiv \overline{\overline{\mathbf{d}}}/L, \quad \overline{\overline{\mathbf{f}}}_{nm}^{Q} \equiv -\frac{\Lambda_n}{\Lambda_m L^2}\lim_{\delta\to 0^+}\oint_{\partial\Omega}\mathbf{E}_n(\mathbf{r}+\delta\hat{\mathbf{n}})\otimes\mathbf{E}_m(\mathbf{r}-\delta\hat{\mathbf{n}})d^2\mathbf{r}.$$

Then, we consider the expression of $\mathcal{H}_{nm}^{R-Q}$:

$$\begin{aligned}\mathcal{H}_{nm}^{R-Q} &= -\lim_{\delta\to 0^+}\frac{\Delta\varepsilon_m}{\varepsilon_{\text{bg}}L^3}\oint_{\partial\Omega}\oint_{\partial\Omega}\left\{\nabla g_{\text{bg}}^{\text{ret}}(\omega;\mathbf{r},\mathbf{r}')\times[\hat{\mathbf{n}}(\mathbf{r})\times\mathbf{E}_n(\mathbf{r}-\delta\hat{\mathbf{n}})]\right\}\cdot\overline{\overline{\mathbf{d}}}(\omega)\cdot\mathbf{E}_m(\mathbf{r}'-\delta\hat{\mathbf{n}}')d^2\mathbf{r}'d^2\mathbf{r}\\ &= -\lim_{\delta\to 0^+}\sum_{k=2,3,\cdots}\frac{k_{\text{bg}}^k\Delta\varepsilon_m}{4\pi\varepsilon_{\text{bg}}L^3}\oint_{\partial\Omega}\oint_{\partial\Omega}\nabla|\mathbf{r}-\mathbf{r}'|^{k-1}\times[\hat{\mathbf{n}}(\mathbf{r})\times\mathbf{E}_n(\mathbf{r}-\delta\hat{\mathbf{n}})]\cdot\overline{\overline{\mathbf{d}}}(\omega)\cdot\mathbf{E}_m(\mathbf{r}'-\delta\hat{\mathbf{n}}')d^2\mathbf{r}'d^2\mathbf{r}.\\ &= -\lim_{\delta\to 0^+}\sum_{k=2,3,\cdots}\frac{(s^R)^k\Delta\varepsilon_m}{4\pi\varepsilon_{\text{bg}}L^{k+2}}\oint_{\partial\Omega}\oint_{\partial\Omega}\nabla|\mathbf{r}-\mathbf{r}'|^{k-1}\times[\hat{\mathbf{n}}(\mathbf{r})\times\mathbf{E}_n(\mathbf{r}-\delta\hat{\mathbf{n}})]\cdot\overline{\overline{\mathbf{s}}}^{Q}\cdot\mathbf{E}_m(\mathbf{r}'-\delta\hat{\mathbf{n}}').\end{aligned} \tag{S.2.9}$$

S3. COMPUTATION SPEED

In this section, it is not our purpose to explicitly compare the computation speed of the present modal method with other methods. Comprehensive comparisons are not easy and generally demand great efforts that are beyond the scope of the present paper. Instead, we intend to show good indicators of computation speed of the present method by refereing to a numerical example. In specifics, we discuss the CPU time needed for computing a bow-tie (with gap size / side length = 1/10, considered in Fig. 4B in the main text) cross-section spectrum on a PC computer equipped with 2.29 GHz×12 processors and a 256 GB memory and with Matlab and COMSOL Multphysics 5.5. The computation times relate to the number of mesh elements used in computing electrostatic plasmon modes and the number of modes retained in the modal method.

Computation of modes.—Electrostatic plasmon modes are solved from Possion's equation

$$\nabla\cdot[\Lambda_n + f(\mathbf{r})]\,\nabla\phi_n(\mathbf{r}) = 0, \tag{S.3.1}$$

where $f(\mathbf{r})$ is a filling function with a value of 1 inside the nanoparticle and 0 otherwise. The eigenvalue problem defined by Equation (S.3.1) is computed with COMSOL Multiphysics. For the studied bow-tie structure, the tetrahedra mesh number is about 2.1×10^4. The computation of four electrostatic modes used in the modal method takes 46 s.

Computation of f factors.—The CPU time for computing the geometric f factors (see Eq. (6) in the main text) is observed to be dominant. Specifically, the computation of $f_{nm;k}^{R}$ and $\overline{\overline{\mathbf{f}}}_{nm}^{R}$ with $n, m = 1, 2, 3, 4$

and $k = 2, 3, 4, 5$, involving double surface integrals, uses 2 mins 21 s. Neverthless, we should note that, once these geometric factors are computed, they can be repeated used for the same geometry even with different sizes and material compositions.

Computation of cross-section spectrum.—The computation of the extinction cross-section for 200 freq. points uses 24.24 s, among which 23.96 s is used to compute the incident field vector $|S\rangle$.

Frequency-domain simulations.—For the numerical results obtained with frequency-domain COMSOL simulations, it takes 50 mins, which is about 14 times longer than the modal method. Again, we emphasize that the speed advantage of the modal method can even be more obvious when more parameters are swept in simulations, e.g., sampling more frequency points or varying polarizations, background media, structure sizes, angles of incident waves.

S4. SUPPLEMENTARY FIGURES

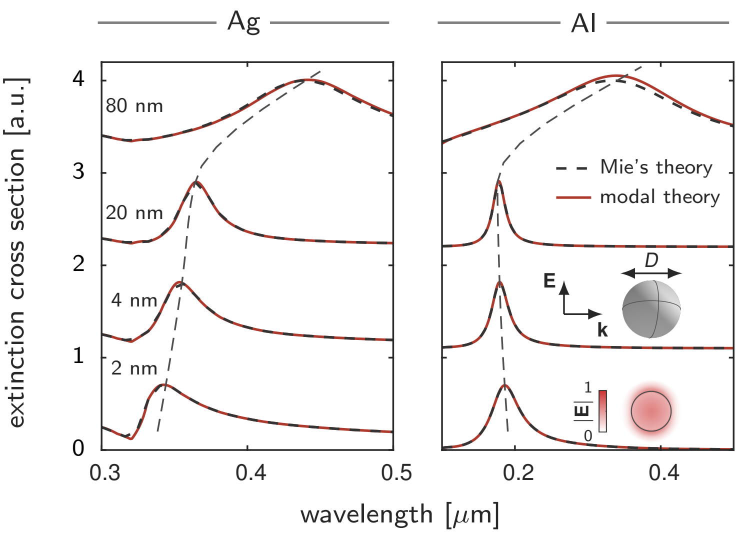

FIG. S1 **Modal analysis of extinction cross-section spectra of metal spheres, contrasting results obtained with the present modal method and Mie's theory.** The Ag and Al nanoparticles are considered, and they are embedded in a dielectric medium with a refractive index of 1.33. The diameter D of the spheres varies from 80 nm to 2 nm. Note that, we only consider TM spherical modes with $\ell = 1$ in Mie's theory to be consistent with the modal method that only uses the dipolar modes (see the inset for the modal profiles). The d-parameters are given in the main text.

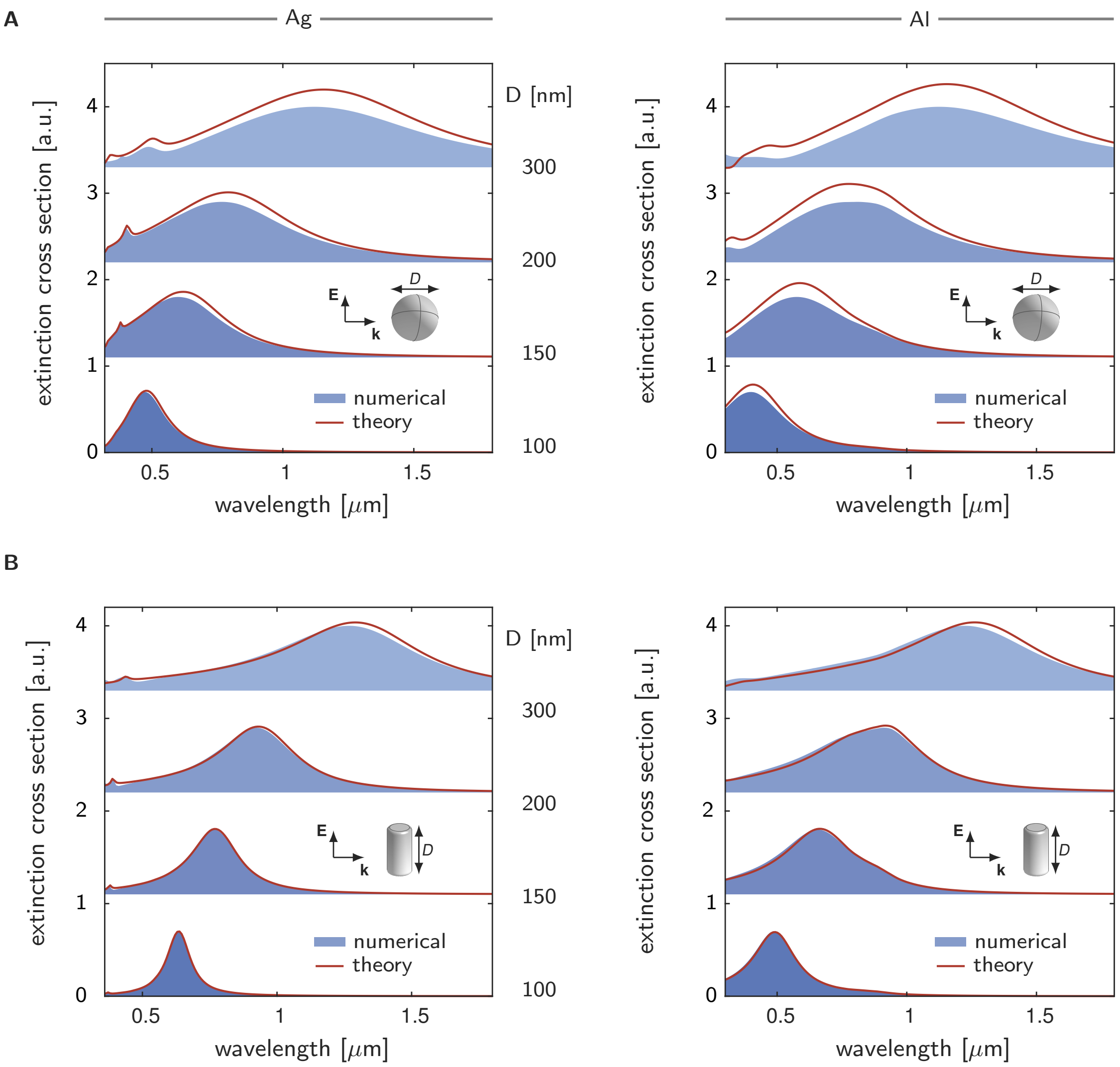

FIG. S2 **Modal analysis of extinction cross-section spectra of metal spheres (A) and rods (B) with large sizes.** The Ag and Al nanoparticles are considered, and they are embedded in a dielectric medium with a refractive index of 1.33. The aspect ratio of the rods (diameter dived by height) in **B** is 0.5. The feature dimension D of the spheres and rods (see the insets for its definition) varies from 100 nm to 300 nm. Note that the agreements between the modal predictions and the numerical solutions obtained with COMSOL Multiphysics degenerate as the particle size increases. In the modal method, a single dipolar mode is retained with its excitation coefficient computed by the independent mode approximation, i.e., Eqs. (7) in the main text. Moreover, in numerical simulations, we only retain the contributions from partial waves with azimuthal order $m = 0$, being consistent with the symmetry of the used dipolar modes.

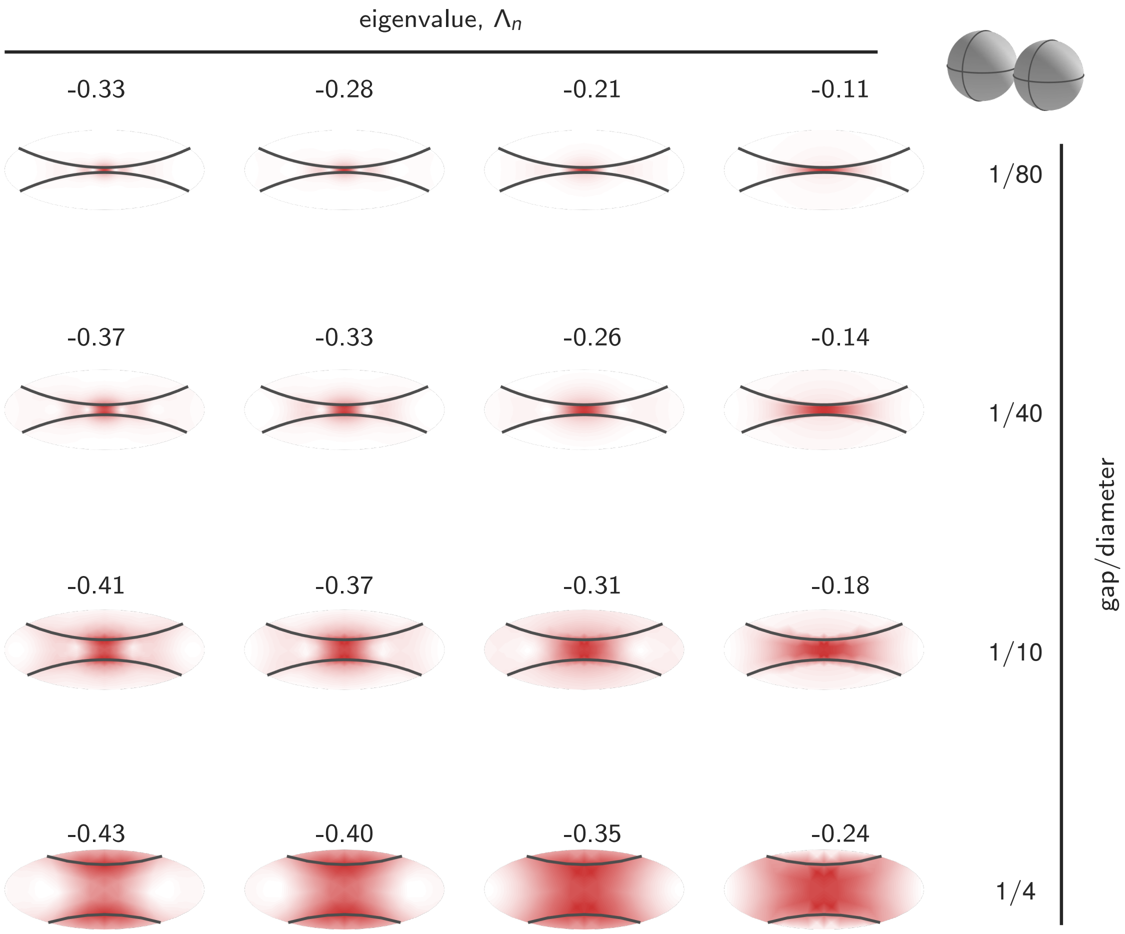

FIG. S3 **Electrostatic surface plasmon modes used for computing optical responses of spherical dimers in Fig. 3A in the main text.**

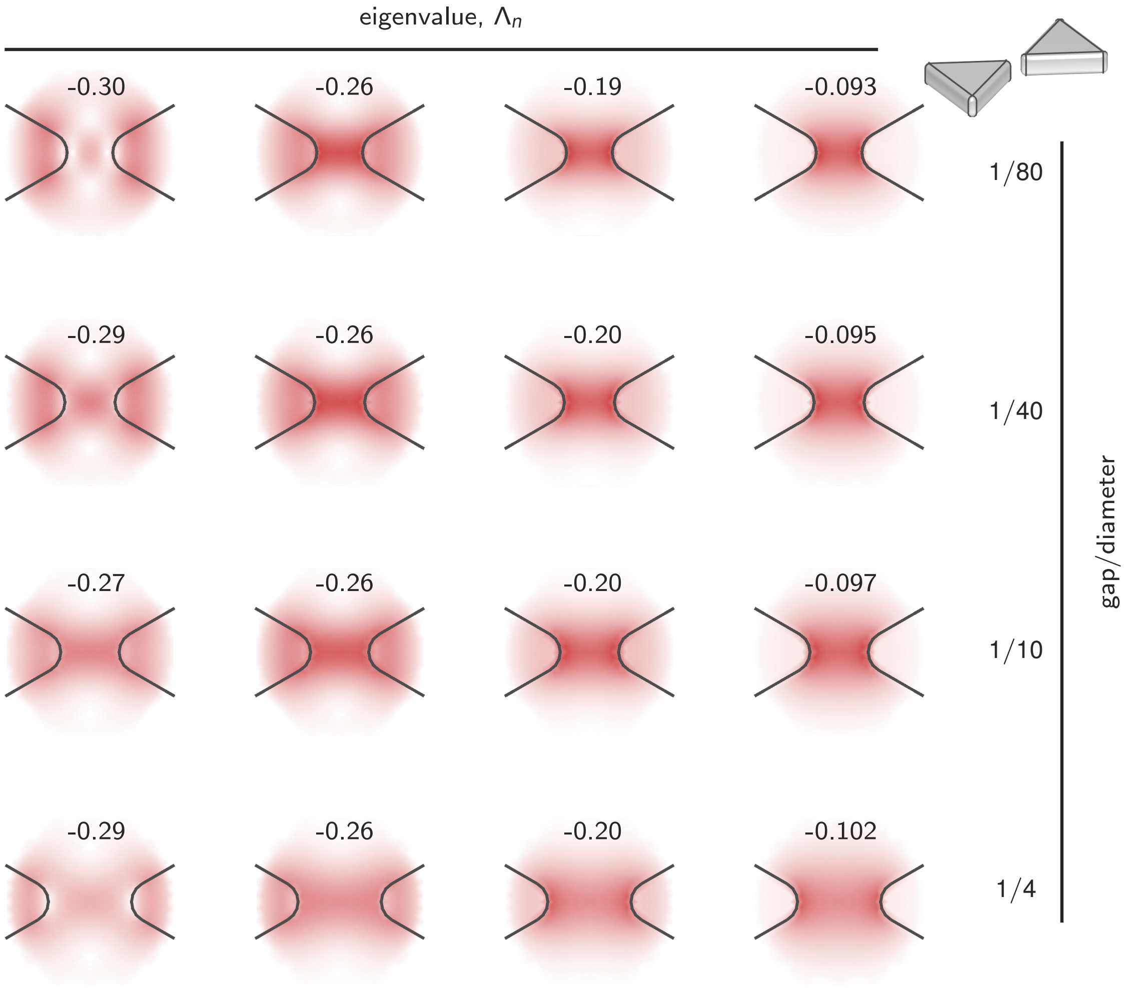

FIG. S4 **Electrostatic surface plasmon modes used for computing optical responses of bow-tie structures in Fig. 3B in the main text.**

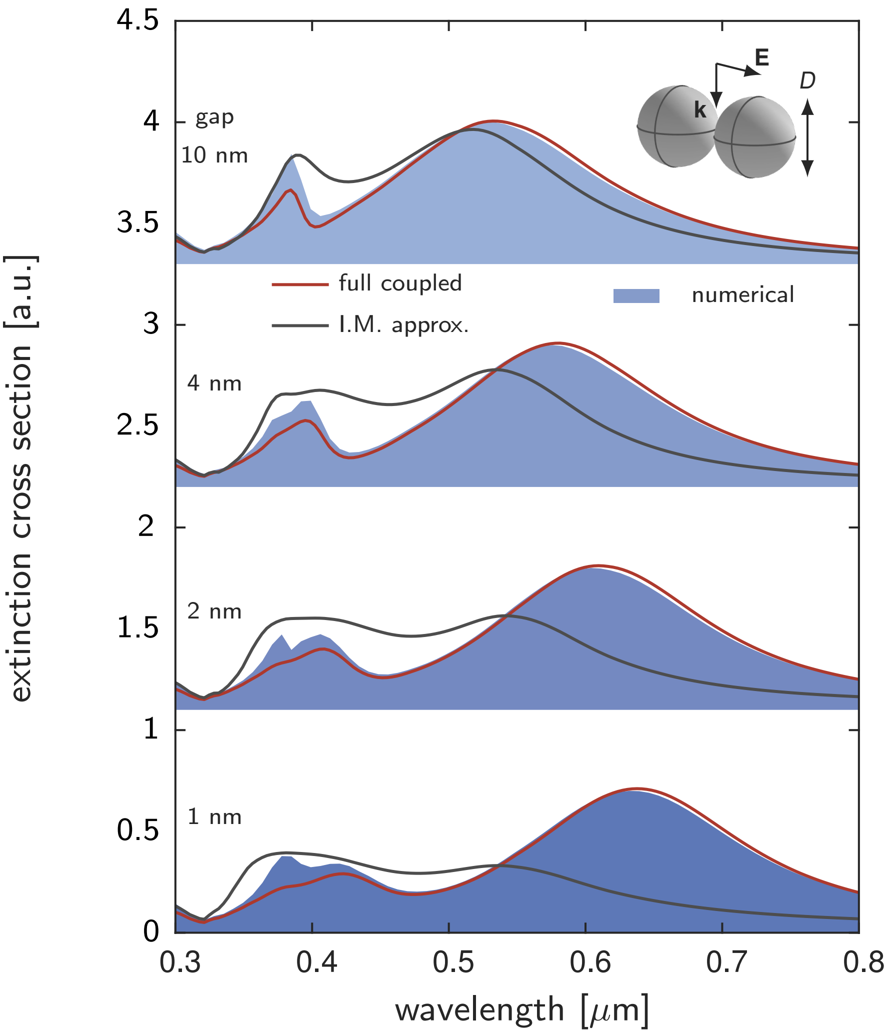

FIG. S5 **Modal analysis of extinction cross-section spectra for sphere dimers, contrasting the results predicted with the independent-mode approximation [I.M. approx.; Eqs. (7) in the main text] and the fully-coupled modal solutions [Eq. (4) in the main text].** The sphere dimers, made of Ag and with a diameter of 80 nm, are embedded in a dielectric medium with refractive index of 1.33. The gap varies from 10 nm to 1 nm. In the modal method, four resonance modes are used (see Fig. S2 for modal profiles). The comparisons between the modal results and the full numerical solutions obtained with COMSOL Multiphysics show that the I.M. approx. fails to produce accurate results, and the inclusion of the modal coupling recovers the numerical accuracy. This observation, together with Fig. 2 and Fig. S1, suggests that, for complex plasmonic structures, such as dimers, the use of the fully-coupled modal method is necessary for the numerical accuracy, while for simple structures, such as single nanoparticles, the simplified I.M. approx. works.

References

* Electronic address: wyanzju@gmail.com
† Electronic address: qiumin@westlake.edu.cn
